# The Effect of Trigger-to-Retrieval Time Interval on Assisted Reproductive Technology Outcomes in Patients with Poor Ovarian Response: The Effect of Trigger-to-Retrieval Time Interval on Assisted Reproductive Technology Outcomes

**DOI:** 10.31661/gmj.v14i.3845

**Published:** 2025-07-02

**Authors:** Fatemeh Sarvi, Masoome Jabarpour, Ashraf Aleyasin, Marzieh Aghahosseini, Sedigheh Hosseini Mousa, Ayda Najafian, Elham Madreseh, Parvin Sadat Eslamnik

**Affiliations:** ^1^ Department of Obstetrics and Gynecology, Shariati Hospital, Tehran University of Medical Sciences, Tehran, Iran; ^2^ Department of Infertility, Shariati Hospital, Tehran University of Medical Sciences, Tehran, Iran; ^3^ Rheumatology Research Center, Tehran University of Medical Sciences, Tehran, Iran; ^4^ Clinical Research Development Unit, Shariati Hospital, Tehran University of Medical Sciences, Tehran, Iran; ^5^ Department of Obstetrics and Gynecology, Emam Sajad Hospital, Yasuj University of Medical Sciences, Iran

**Keywords:** Dual Trigger, Oocyte Pickup (OPU), Poor Ovarian Response (POR), Assisted Reproductive Technology (ART)

## Abstract

**Background:**

The timing of oocyte retrieval after the oocyte maturation trigger is a critical factor influencing the clinical outcomes of assisted reproductive technologies (ART). This study examined how different time intervals of 34 and 36 hours between trigger administration and oocyte pickup (OPU) influence on ART outcomes in patients with poor ovarian response (POR).

**Materials and Methods:**

This prospective randomized controlled study enrolled 217 women undergoing intracytoplasmic sperm injection (ICSI) cycles between April 2024 and March 2025. All participants received a GnRH antagonist protocol, followed by a dual trigger for final oocyte maturation. The cycles were stratified into two groups based on the time interval between trigger administration and OPU at 34 and 36 hours. Comparative analyses were conducted to evaluate the outcomes between the two groups.

**Results:**

Throughout the trial, no significant statistical differences were observed between the intervention and control groups concerning baseline clinical and demographic characteristics, except for the antral follicle count (AFC) at baseline. The findings revealed that the 34-hour OPU group exhibited a significantly higher number of retrieved oocytes, oocyte maturity rate, fertilization rate, and number of high-quality embryos compared to the 36-hour group (P0.05). Furthermore, pregnancy outcomes were most favorable in the 34-hour group (P0.05). After adjusting for AFC, all observed differences remained statistically significant, with the exception of the fertilization rate.

**Conclusions:**

Our findings suggest that in POR patients, a 34-hour OPU interval with dual triggering significantly improves fertilization rates, embryo quality, biochemical and chemical pregnancy rates compared to 36 hours. This timing adjustment may enhance oocyte maturation and optimize ART success rates.

## Introduction

In assisted reproductive technology (ART), the goal of controlled ovarian stimulation (COS) is to promote the development of multiple dominant follicles and support oocyte maturation, ultimately increasing the chances of conception. Throughout this process, oocytes undergo both nuclear and cytoplasmic maturation, which are essential for maximizing fertilization potential and developmental competence [1][2]. COS involves two key time points: (i) ovulation triggering and (ii) oocyte retrieval. The period between these stages is crucial for in vivo oocyte maturation, which is regulated by a complex cascade of biochemical processes [3][4]. The interval between ovulation trigger and oocyte pickup (OPU) is crucial, as it encompasses the initiation of luteinization, the expansion of cumulus cells, and the resumption of oocyte meiosis through reduction division [5]. Several studies have indicated that the interval between hCG administration and OPU significantly influences follicular maturation, the proportion of oocytes with fully expanded cumulus, the number of metaphase II (MII) oocytes, embryo developmental potential, and overall IVF outcomes [5][6][7][8]. The timing of oocyte retrieval following the oocyte maturation trigger plays a crucial role in determining the clinical outcomes of ART. However, findings on the optimal duration of this interval remain inconsistent. While some studies have reported that extending the time to OPU does not necessarily lead to a higher yield of mature oocytes or improved clinical outcomes [9][10][11][12], others have suggested that a prolonged interval may enhance oocyte maturation[13][14][5], increase fertilization rates [15], improve blastocyst development [16], result in a greater number of high-quality embryos, and contribute to higher ongoing pregnancy rates [17]. Additionally, some studies have indicated that extending the interval between human chorionic gonadotropin (hCG) administration and oocyte retrieval predominantly increases the proportion of metaphase II (MII) oocytes without significantly impacting pregnancy rates[18][19]. To date, there is no consensus regarding the ideal hCG-OPU interval, with reported durations in clinical practice ranging from 32 to 38 hours [13][12]. Limited research exists on the influence of trigger-to-OPU intervals in patients with poor ovarian responses patients, despite studies focusing on the general in vitro fertilization (IVF) population. Due to their low oocyte yield and suboptimal quality, these patients may be highly affected by even slight variations in timing. A recent study demonstrated that in patients with diminished ovarian reserve (DOR), prolonging the interval between hCG administration and oocyte retrieval to 36 hours led to enhanced fertilization rates and improved embryo development compared to intervals of 34 and 35 hours. Nevertheless, this extension did not influence pregnancy outcomes [20]. Determining the optimal timing for oocyte retrieval to improve ART outcomes continues to be a complex issue, particularly among poor ovarian responders—a population that is often underrepresented in current research. This study seeks to investigate the effect of the interval between the dual trigger for final oocyte maturation and oocyte retrieval on ART outcomes in this specific group. Given the limited data addressing this relationship in poor responders, the findings of this research may offer valuable insights. By exploring associations between treatment variables and timing intervals, the study aims to inform more effective scheduling protocols within ART practices.

## Materials and Methods

### Trial Design

This study is designed as a randomized, single-blind clinical trial in which patients are blinded to the type of intervention. The trial will be conducted 217 infertile patients from April 2024 to March 2025 at the infertility clinic of Shariati Hospital, affiliated with Tehran University of Medical Sciences (TUMS), Tehran, Iran. This registered prospective randomized controlled trial (IRCTID: IRCT20120215009014N512) was approved by the Institutional Ethical Review Board (IR.TUMS.SHARIATI.REC.1403.012). Prior to enrollment, all participants were provided with a detailed explanation of the study's objectives and procedures, and written informed consent was obtained. Those who met the inclusion criteria and provided consent were subsequently enrolled in the study. The inclusion criteria are based on the Bologna or POSEIDON III and IV definitions for poor ovarian responders. Poor ovarian response is diagnosed based on advanced maternal age (≥40 years), a history of poor response in prior cycles (≤3 oocytes or cycle cancellation), or abnormal ovarian reserve tests (AFC ≤5-7 or AMH ≤1.1 ng/ml). Meeting at least two of these criteria confirms the diagnosis. Participants will be were excluded if they meet met any of the following conditions: Stage III or IV endometriosis, History of ovarian surgery, Requirement for preimplantation genetic diagnosis (PGD), Sever Male factor (TESE). After enrollment, all participants will undergo a standard treatment cycle. All cycles that were canceled in the study occurred after patient enrollment, either due to the cancellation of the embryo transfer cycle or the OPU cycle (such as ovulation occurring before puncture or empty follicle syndrome). During the initial evaluation of participants, a transvaginal ultrasound was conducted to assess uterine and ovarian normalcy, while a semen analysis was performed to evaluate sperm parameters, including count, morphology, and motility. Furthermore, hormonal profiling was undertaken on days 1-3 of the menstrual cycle, measuring levels of thyroid-Stimulating Hormone (TSH) TSH, FSH, AMH, vitamin D, LH, estradiol, and prolactin to ensure baseline physiological normality.

### COS Protocol and Randomization

If no abnormalities are identified during the initial evaluation, patients proceed to the treatment cycle, which begins on the second or third day of menstruation, as determined by the attending physician. The treatment involves the administration of the maximum dose of gonadotropins within an antagonist protocol. On the seventh day of stimulation, a transvaginal ultrasound is conducted to monitor follicular development. When the follicles reach a size of 13-14 mm, a daily subcutaneous injection of GnRH antagonist (cetrorelix 0.25 mg) is initiated. A follow-up ultrasound is performed 2-3 days later, and if at least three dominant follicles with a diameter of ≥18 mm is observed, oocyte maturation is triggered using a dual injection of highly purified urinary hCG (10,000 IU) and GnRH agonist (Decapeptyl 0.2 mg). Participants were randomly assigned to one of two groups using a simple randomization method implemented in R (version 4.4.1) software. The control group undergoes oocyte retrieval 36 hours after the trigger injection, while the intervention group undergoes the procedure 34 hours post-trigger. Patients were blinded to the type of intervention and they were assigned to groups based on a random sequence available to the researcher. In both groups, follicle aspiration and oocyte retrieval are performed under general anesthesia with transvaginal ultrasound guidance. All retrieved metaphase II (MII) oocytes are fertilized through intracytoplasmic sperm injection (ICSI). Subsequently, based on progesterone levels on the trigger day, embryo quality, and endometrial thickness, the resulting embryos are either cryopreserved or prepared for fresh transfer.

### Clinical Data

After denudation, the retrieved oocytes were evaluated for quality and maturity, categorized as germinal vesicle (GV), metaphase I (MI), or metaphase II (MII). ICSI was performed on the MII oocytes, and fertilization was assessed 16-18 hours later by the presence of two pronuclei (2PN). Reproductive outcomes were measured, including the number of oocytes retrieved, the number of MII oocytes, and the oocyte maturity rate (percentage of normal MII oocytes out of total retrieved normal oocytes). The fertilization rate was calculated by dividing the number of oocytes with 2PN (16-18 hours post-insemination) by the number of MII oocytes injected. High-quality embryos were defined as grade A and B cleavage embryos according to ASEBIR criteria. Additionally, the chemical pregnancy rate (positive β-hCG test 14 days after embryo transfer, expressed as a percentage of ET cycles), and the clinical pregnancy rate (evidence of pregnancy via ultrasound 5-6 weeks after ET, expressed as a percentage of ET cycles) were recorded.

### Endometrial Preparation

In the frozen embryo transfer (FET) protocol, estradiol (E2) was administered on days 2-3 of the cycle, either orally or vaginally, with monitoring of endometrial thickness by ultrasound after 10 days. If the endometrium reached ≥7 mm with a trilaminar pattern, progesterone therapy was initiated. If not, estradiol was continued until the endometrium met the required thickness. Once optimal, progesterone (50 mg intramuscularly and 800 mg vaginally) was started, and embryos were transferred on day 4 for cleavage-stage embryos. In stimulated cycles for embryo freezing, letrozole (5 mg) was administered from day 2, with ultrasound assessments on day 12. If a dominant follicle reached ≥14 mm and endometrial thickness was ≥7 mm, a follow-up ultrasound occurred 3 days later. If the follicle reached 18-20 mm, HCG was administered, and embryo transfer occurred 7 days later. In fresh embryo transfer cycles, progesterone (50 mg intramuscularly and 800 mg vaginally) was started post-ovulation, with embryo transfer occurring on day 4 for cleavage-stage embryos. IUI was performed in the operating room for patients with no available oocytes.

### Primary and Secondary Outcomes

The primary outcome of this study is the oocyte maturity rate. Secondary outcomes include the total number of oocytes retrieved, the number of mature oocytes (MII), the fertilization rate, the number and quality of embryos, as well as the biochemical pregnancy rate and clinical pregnancy rate.

### Sample Size and Statistical Analysis

The oocyte maturity rate was identified as the primary outcome. Based on the findings of Raziel et al. (2006) [7] and accounting for a 6% loss to follow-up, the required sample size was determined to be 202 participants (101 per group) to achieve 90% statistical power with a significance level of 0.05 for detecting differences in oocyte maturity rate. Data analysis was conducted using IBM SPSS Statistics version 24 . The Kolmogorov-Smirnov test was used to assess the normality of data distribution. Qualitative variables were presented as frequency and percentage, while quantitative data with a normal distribution were expressed as mean ± standard deviation. Differences between the placebo and treatment groups were evaluated using the Mann-Whitney U test, Fisher’s exact test, or the Chi-square test, with statistical significance set at P<0.05. Additionally, linear regression and multiple logistic regression models were applied to adjust for potential confounders (AFC day 3). In this study, a pre-protocol approach was adopted.

## Results

**Table:** Table1. Baseline Characteristics of Study Participants

**Variables**		**Groups **		**P-value ^+^**
**Intervention ** **n=109** **Control** **n=108**				
Age (years)		36.96 ± 4.73	37.55 ± 4.33	0.49
BMI (kg/m^2^)		27.04 ± 3.33	27.22 ± 4.07	0.909
Infertility. Type (years)	PIF	82 (75%)	76 (70%)	0.448
	SIF	27 (25%)	32 (30%)	
Duration of infertility (years) PIF SIF 6.14 ± 4.87 6.26 ± 4.79 6.31 ± 4.46 6.23 ± 5.04		0.827 0.748		
Menarche Age (years)		13.35 ± 1.44	13.10 ± 1.32	0.063
Menstruation days		5.55 ± 1.4	5.44 ± 1.20	0.482
IVF History (N)_		1.24 ± 1.05	1.20 ± 1.27	0.426
Baseline FSH (IU/ml)		8.60 ± 4.78	9.24 ± 6.27	0.780
Baseline E2 (pg/dL)		56.59 ± 39.07	83.77 ± 99.38	0.142
Baseline LH (IU /ml)		5.85 ± 4.22	5.32 ± 3.27	0.826
Baseline PRL (ng/ml)		5 (4.6%)	10 (9.3%)	0.193
AMH (ng/ml)		0.77 ± 0.28	0.74 ± 0.33	0.833
VitD3 (ng/ml))		33.72 ± 14.09	31.47 ± 13.56	0.270
TSH (MIU/L)		2.14 ± 1.23	2.32 ± 1.15	0.215
AFC day3 (N)		5.02 ± 1.56	4.60 ± 1.03	**0.036**
Total gonadotropin dose (IU)		4043 ± 1439	3850 ± 1662	0.36 ^++^
Gonadotropin Administration Period (Days)		10.78 ± 2.06	11.06 ± 2.11	0.205
No. of fresh embryo transfer cycle		52 (48%)	38 (35%)	0.061
No of frozen embryo transfer cycle		46 (42%)	33 (31%)	0.075
Abnormal Uterine Findings on HSG (N)		30(27.5%)	18(16.7%)	0.057
	OPEN (normal)	81(74.3%)	84(77.8%)	
Tubal Findings on HSG (N)	CLOSE	17(15.6%)	11(10.2%)	0.494
	hydrosalpinx	5(4.6%)	6(5.6%)	
Abnormal Hysteroscopy (N)		29(26.6%)	21(19.4%)	0.486

+Calculated based on Mann–Whitney or Fisher exact or chi-square tests. ++Calculated based on t-test. ^*^ Because the missing (not reported) percentage is not equal to 100%

**Table:** Table2. Comparison ART Outcomes between Two Groups

**Variables**	**Groups **		**P-value ^+^**	**P-value ^++^**
	**Intervention ** **n=109**	**Control** **n=108**		
Oocyte. No.	4.78 ± 2.48	2.50 ± 1.94	**< 0.001**	**< 0.001**
Oocyte Retrieval Rate%	90.93 ± 15.91	48.94 ± 28.53	**< 0.001**	**< 0.001**
MII Oocyte No.	3.46 ± 1.91	1.80± 1.53	0.098	0.107
Oocyte Maturity Rate%	72.46 ± 22.02	64.07 ± 38.03	**0.005**	**0.040**
Fertilization Rate%	91.73 ± 26.48	70.54 ± 45.56	**0.001**	0.161
OPU cancellation Rate% (Ovulated or EFS)	1(1%)	25 (23%)	**< 0.001**	**< 0.001**
ET cancellation Rate%	10 (9%)	12 (11%)	0.636	0.792
Cancellation Rate% (OPU + ET)	11 (10%)	37 (34%)	**< 0.001**	**< 0.001**
No. of ETs per patient*	2.19 ± 0.71	1.68 ± 0.71	**< 0.001**	**< 0.001**
Total Transferred Embryos (by quality) *				
A	29.76±36.63	11.97±31.14	**< 0.001**	**0.002**
B	69.7±36.56	86.39±32.03	**< 0.001**	**0.005**
C	0.51±5.05	1.64±9.82	0.380	0.325
Biochemical Preg*	37 (37.8%)	13 (18.3%)	**0.007**	**0.015**
Clinical Preg*	31 (31.6%)	10 (14.1%)	**0.010**	**0.016**

+Calculated based on Mann–Whitney. ++ Adjusted in linear or logistic regression models by AFC day3.^*^ Among 169 patients. ET embryo transfer

**Figure-1:**
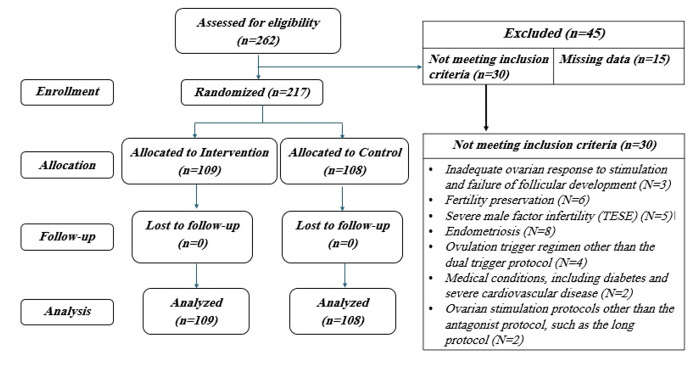
Figure 1. Summary of patient flow through the study

As illustrated in Figure-1, the study included a total of 217 patients, with 109 in the intervention group and 108 in the placebo group. Throughout the trial, no significant statistical differences were observed between the intervention and control groups regarding infertility duration, average age, BMI, hormonal profile, and other baseline clinical and demographic factors—except for AFC at the study's outset (Table-1). The analysis accounted for the effect of AFC. Table-2 demonstrates that the intervention group had significantly higher oocyte numbers (4.78 vs. 2.50, P<0.001) and oocyte retrieval rate (90.93% vs. 48.94%, P<0.001) compared to the control group. Similarly, the oocyte maturity rate was greater in the intervention group (72.46% vs. 64.07%, P=0.005), though the number of MII oocytes showed no significant difference between groups (3.46 vs. 1.80, P=0.098). Additionally, the intervention group had a significantly higher fertilization rate (91.73% vs. 70.54%, P<0.001). In addition, the cancellation rate (OPU + ET) was notably higher in the control group (P<0.001). Regarding embryos, both the number of embryos per transfer and the proportion of high-quality embryos (A and B) were significantly greater in the intervention group (P<0.001). Clinical pregnancy rates (P=0.01) and biochemical pregnancy rates (P=0.007) were also significantly higher in the intervention group. After adjusting for AFC, changes in all variables remained significant, except for the fertilization rate.

Additionally, we compared the variables between the two groups, categorized by the transfer cycle (fresh or frozen embryo transfer) (Table-3) and the endometrial preparation protocol in frozen embryo transfer (Table-4). In frozen embryo transfer cycles, both the number of embryos per transfer and the proportion of high-quality embryos (A and B) were significantly higher in the intervention group (P<0.001). In fresh embryo transfer cycles, this was only true for the number of embryos per transfer (P=0.02). Furthermore, in fresh embryo transfer cycles, biochemical pregnancy rates (P=0.035) and clinical pregnancy rates (P=0.058) were also significantly higher in the intervention group. As shown in Table-5, in HRT cycles, the number of embryos per transfer was significantly higher in the intervention group (P=0.047). However, in stimulated cycles, the number of high-quality embryos (A and B) was significantly higher in the intervention group (P=0.046).

## Discussion

**Table:** Table3. Comparison of Variables between the Two Groups, Stratified by Transfer Cycle

**Variables**			**Fresh transferred ** **n=90 ( 53.3%)**			**Freeze transferred ** **n=79 ( 46.7%)**	
		**Intervention ** **n=52 (57.8%) **	**Control** **n=38 (42.2%) **	**P-value+**	**Intervention** **n=46 (58.2%) **	**Control** **n=33 (41.8%) **	**P-value+**
No. of ETs per patient *		2.19±0.63	1.61±0.64	**< 0.001**	2.20±0.81	1.76±0.79	**0.020**
	A	32.05±35.98	6.58±23.74	**< 0.001**	27.17±37.57	18.18±37.35	0.127
Total Transferred Embryos (by quality)	B	66.99±35.77	91.67±25.63	**< 0.001**	72.83±37.57	80.30±37.61	0.207
	C	0.96±6.93	1.75±10.81	0.810	00.00±NM	1.51±8.70	0.238
Biochemical Preg*		14 (26.9%)	5 (13.2%)	0.127	23 (50.0%)	8 (24.2%)	**0.035**
Clinical Preg*		12 (23.1%)	3 (7.9%)	0.085	19 (41.3%)	7 (21.2%)	**0.058**

+Calculated based on Mann–Whitney or Fisher exact tests. ^*^ Only among transfer patients.**NM:** not meaning

**Table:** Table4. Comparison of Variables between the Two Groups based on Endometrial Preparation Cycle in Frozen Embryo Transfer

**Variables**					**Freeze transferred ** **n=79**		
		**HRT** **n=71 ( 89.9%)**			**Simulated** **n=8 (10.1%) **		
		**Intervention ** **n=42 ( 59.2%)**	**Control** **n=29 (40.8%) **	**P-value ^+^**	**Intervention** **n=4 (50.0%) **	**Control** **n=4 (50.0%) **	**P-value ^+^**
Endometrial preparation period		17.79 ± 2.04	17.79 ± 1.74	0.848	17.50 ± 2.38	17.75 ± 2.75	0.882
Endometrial thickness		9.67 ± 1.95	9.40 ± 1.32	0.573	8.95 ± 0.70	9.23 ± 2.02	0.773
No. of ETs per patient		2.19 ± 0.80	1.79 ± 0.82	**0.047**	2.25 ± 0.96	1.50 ± 0.58	0.222
	A	24.21±35.54	20.69±39.25	0.386	58.33±50.00	00.00±NM	**0.046**
Total Transferred Embryos (by quality)	B	75.79±35.54	77.59±39.41	0.559	41.67±50.00	100.00±NM	**0.046**
	C	00.00±NM	1.72±9.28	0.229	00.00±NM	00.00±NM	0.999
Biochemical Preg		21 (50.0%)	8 (27.6%)	0.086	2 (50.0%)	0 (0.0%)	0.429
Clinical Preg		18 (42.9%)	7 (24.1%)	0.133	1 (25.0%)	0 (0.0%)	0.429

+Calculated based on Mann–Whitney or Fisher exact tests.**NM:** not meaning

In current randomized clinical trial, we examined the impact of the interval between dual trigger (hCG and decapeptyl) administration and oocyte retrieval on oocyte maturation and ART outcomes in POR. Our findings indicate that a shortened interval (34 hours) between dual trigger (hCG and decapeptyl) administration and oocyte retrieval results in significantly higher oocyte yield, oocyte retrieval rates, MII oocyte numbers, fertilization rates, number of high-quality embryos, and overall improved ART outcomes compared to the conventional 36-hour interval.

The timing between trigger and OPU is critical for the success of ART, as processes such as luteinization, cumulus cell expansion, and resumption of meiosis must occur before aspiration [21]. To achieve optimal outcomes, precise management of this interval is essential to ensure a higher yield of mature oocytes while preventing spontaneous ovulation [22]. Physiological studies suggest that ovulation generally takes place between 24 and 56 hours following the LH surge, with an average occurrence at 32 hours [23]. Nader and Berkowitz [21] suggested that ovulation may occur earlier than 36 hours in some women, advising that intervals under 35 hours should be targeted to prevent ovulation.

There is limited research on the impact of trigger-to-OPU intervals in patients with poor ovarian response, despite studies conducted on the general IVF population, which have yielded conflicting results. A recent study showed that in patients with diminished ovarian reserve (DOR), extending the interval between hCG administration and oocyte retrieval to 36 hours resulted in improved fertilization rates and better embryo development compared to 34- and 35-hour intervals. However, this extension did not affect pregnancy outcomes [20]. Their findings revealed no statistically significant differences between the groups regarding biochemical pregnancy rate (P=0.252), clinical pregnancy rate (P=0.867), total pregnancy loss rate (P=0.859), or live birth rate (P=0.338). Although there was a trend toward higher biochemical and clinical pregnancy rates in the 36-hour OPU group, the lack of statistical significance suggests that variations in OPU timing may not substantially affect pregnancy outcomes. Consistent with their results, Wang et al.[19] found no significant impact of OPU timing on pregnancy rates. However, other studies have reported improved pregnancy outcomes with later OPU timings [10][24][7].

Wei Wang et al. conducted a meta-analysis including five RCTs with 895 participants, which showed that the oocyte maturation rate was higher in the long interval group (>36 hours) compared to the short interval group (<36 hours) [19]. However, the findings differed from our study, likely due to the inclusion of patients with low ovarian reserve in our cohort. Similarly, Runxin Gan et al.'s meta-analysis reported comparable maturation rates in both the short and long interval groups (85.6% and 87.4%, respectively) [23]. These results also contrasted with ours, as their study focused on patients with polycystic ovary syndrome (PCOS), while our study specifically targeted patients with poor ovarian reserve.

Inconsistent with our study's findings, Garor et al. [10] found that delayed OPU was associated with a greater number of embryos and higher fertilization rates compared to early OPU. A key finding from their study was the significantly higher OPU cancellation rate due to early ovulation in the 34-hour OPU group (15.7%) compared to the 35-hour (3.5%) and 36-hour (2.2%) groups (P<0.001). To minimize the risk of premature ovulation, the 34-hour OPU group had its OPU procedure scheduled earlier due to the elevated LH levels on the trigger day. However, early OPU did not prevent premature ovulation in this group, resulting in fewer mature oocytes and lower fertilization rates. Similarly, Choi et al. [25] also reported that early oocyte retrieval during an early LH surge did not effectively reduce cycle cancellation rates and may contribute to lower fertilization rates.

In line with previous studies, Skvirsky et al. [26] demonstrated that extending the interval between hCG administration and OPU could enhance oocyte maturation and embryo quality in women over 36 years of age. The blastocyst formation rate differed significantly among the groups (P=0.025), with the highest rate observed in the 36-hour OPU group. This suggests that delaying OPU to 36 hours may benefit blastocyst development, potentially due to improved oocyte maturity and cytoplasmic competence. These results imply that a longer interval between trigger and OPU may improve embryo quality at later stages.

The discrepancies between our study and prior research may be attributed to key factors such as differences in study populations (POR vs. DOR or PCOS patients or general IVF patients), triggering protocols (dual trigger vs. hCG-only trigger), and Ovarian Physiology (POR patients may need earlier OPU to prevent over-maturation and loss of viable oocytes). Our findings highlight that for POR patients, a shorter trigger-to-OPU interval enhances ART success rates, which may serve as an important consideration in refining individualized stimulation protocols for this specific subgroup of patients.

This study offers a novel approach to improving ART outcomes in POR patients by optimizing OPU timing with dual triggering, which enhances fertilization rates, embryo quality, and pregnancy outcomes. The inclusion of POR patients strengthens its clinical relevance, as this population faces significant challenges in ART. Additionally, the RCT and prospective design enhance the study’s validity by reducing bias and providing a clear assessment of causality. However, limitations include a small sample size, lack of long-term follow-up on live birth rates, and a single-center design, which may limit the generalizability of the findings. Further studies with a larger, more diverse cohort are needed to validate these results and refine ART protocols.

## Conclusion

Our findings suggest that in patients with POR, a 34-hour interval between dual triggering and OPU, significantly improves fertilization rates, embryo quality, and pregnancy outcomes when compared to the standard 36-hour interval. This timing adjustment appears to optimize the maturation of oocytes, leading to higher-quality embryos, which, in turn, may enhance the likelihood of successful fertilization and pregnancy. By fine-tuning the timing of OPU, especially in women with POR, we may improve the overall success rates of ART, offering a more effective approach to fertility treatment for this specific patient population.

## Conflict of Interests

The authors declare no conflicts of interest.
